# Immediate effect of quadri-pulse stimulation on human brain microstructures and functions

**DOI:** 10.1162/imag_a_00264

**Published:** 2024-08-12

**Authors:** Ikko Kimura, Masamichi J. Hayashi, Kaoru Amano

**Affiliations:** Center for Information and Neural Networks (CiNet), Advanced ICT Research Institute, National Institute of Information and Communications Technology, Suita, Japan; Graduate School of Frontier Biosciences, Osaka University, Suita, Japan; Graduate School of Information Science and Technology, The University of Tokyo, Tokyo, Japan

**Keywords:** quadri-pulse stimulation, repetitive transcranial magnetic stimulation, diffusion magnetic resonance imaging, fractional anisotropy, mean diffusivity, plasticity

## Abstract

It remains unclear whether repetitive stimulation of a single brain area immediately alters brain microstructure. Thus, we investigated the immediate changes in human brain microstructures following repetitive extrinsic excitation of the left primary motor cortex (M1) through quadri-pulse stimulation (QPS). Sixteen right-handed healthy adults underwent excitatory (QPS5) and inhibitory (QPS50) QPS. Diffusion magnetic resonance imaging (MRI) and resting-state functional MRI were conducted before and after QPS to detect microstructural and functional changes, respectively. No significant alterations in microstructural indices after QPS5 or QPS50 were observed in the cerebral cortex. The functional connectivity (FC) between the bilateral M1 was significantly decreased after QPS5, while it was not significantly modulated after QPS50. Microstructural changes exhibited no significant correlation with this FC change in any region after QPS5 or QPS50. Although no significant FC change was observed following QPS50, these results may suggest that repetitive stimulation of a single brain area can be insufficient to induce immediate microstructural alterations. This would be supported by demonstrating the lack of microstructural changes after QPS together with changes in cortical excitability of the stimulated region.

## 1 Introduction

Microstructures in the brain alter after repeated brain activation induced by behavioral training and brain stimulation. This process is mediated by long-term plasticity, which modulates both function and structure of the brain (Ugawa, 2012). Repeated stimulation of the presynaptic neuron increases the number of dendritic spines (Engert & Bonhoeffer, 1999) and induces neurogenesis (Leal et al., 2014) and angiogenesis (Licht et al., 2011). It also evokes microstructural plasticity in glial cells near the postsynaptic neurons, such as astrocytes (Sagi et al., 2012) and myelin (Gibson et al., 2014) (seeCullen & Young (2016)for review). Non-invasive brain imaging studies have suggested that these microstructural modifications can also occur in the adult human brain after behavioral training (Kodama et al., 2023;Sagi et al., 2012;Sampaio-Baptista et al., 2021;Scholz et al., 2009) and non-invasive brain stimulation (Jung & Lambon Ralph, 2021;Lazari et al., 2022;May et al., 2007). However, the boundary condition for such microstructural modifications remains unclear.

While microstructural modifications in the brain have been proposed to require hours to days (Ugawa, 2012), evidence from several studies suggests that these alterations can also occur immediately after behavioral training (Johansen-Berg et al., 2012). Sagi et al. demonstrated that microstructural properties revealed by diffusion magnetic resonance imaging (dMRI) were modulated immediately after a single session of a 2-hr spatial memory task (Sagi et al., 2012). Furthermore, a recent study has demonstrated that microstructural changes can be observed immediately after a 45-min motor training (Tavor et al., 2020). Understanding these ultra-acute microstructural alterations is also vital for unraveling the relationship between microstructural plasticity and functional activity. Nevertheless, it remains unclear whether the microstructure also undergoes immediate changes following the neural plasticity induced by simple repetitive excitations within a single brain region as behavioral training activates widespread areas (Bueichekú et al., 2019).

Although repetitive transcranial magnetic stimulation (rTMS) is a well-established method to either enhance or suppress brain activity of a single region, depending on the protocol, only a few previous studies have investigated the immediate microstructural changes following rTMS. These studies, focusing on excitatory (Niehaus et al., 2000) or inhibitory (Abe et al., 2014;Duning et al., 2004;Mottaghy et al., 2003) protocols of conventional rTMS, have generated inconsistent results. These inconsistencies might stem from large inter-individual variability in plasticity induced by conventional rTMS (Maeda et al., 2000;Prei et al., 2023). In contrast, quadri-pulse stimulation (QPS;Hamada et al., 2008;Kimura et al., 2022), a patterned rTMS protocol, relatively robustly modulates the brain activity of the stimulated region across participants. While the excitatory protocol of QPS (QPS5) over the primary motor cortex (M1) is reported to enhance the motor-evoked potential (MEP) in 86% of participants, the inhibitory protocol of QPS (QPS50) is reported to suppress the MEP in 94% of participants (Nakamura et al., 2016), which was replicated in our own study (Kimura et al., 2022). Other studies have reported that the MEP change after QPS over M1 was stronger compared to that following other rTMS protocols, such as paired associative stimulation (Hamada et al., 2007) and theta-burst stimulation (Tiksnadi et al., 2020).

Therefore, in this study, we employed QPS to examine whether the microstructural properties undergo immediate alterations following QPS5 and QPS50, and whether the degree of change differs between QPS5 and QPS50. Additionally, we evaluated the change in functional connectivity (FC) of the stimulated region after QPS to clarify the relationship between microstructural and functional changes. This knowledge is crucial for clarifying the susceptibility of structural plasticity in the ultra-acute phase and elucidating the potential of rTMS as a method for modulating the microstructural properties of the human brain.

## 2 Methods

**Fig. 1.**
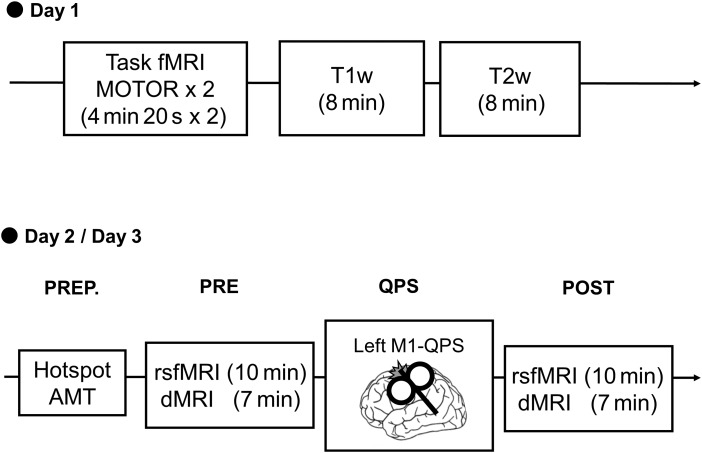
Overview of the experimental protocol to assess the microstructural changes after quadri-pulse stimulation (QPS) over the left primary motor cortex (M1). On Day 1, task functional magnetic resonance imaging (Task fMRI) data were acquired during a finger-tapping task (MOTOR) to localize the left M1, whereas T1-weighted (T1w) and T2-weighted (T2w) images were obtained for neuronavigation of the TMS coil and for spatial normalization and surface-based analysis of the diffusion MRI (dMRI) and resting-state functional MRI (rsfMRI) data. On Days 2 and 3, QPS5 or QPS50 was performed. First, in the preparation step (PREP.), the hotspot for the left M1 and the active motor threshold [AMT] were defined to determine the stimulus location and intensity (90% of AMT), respectively. Participants underwent resting-state fMRI (rsfMRI) and diffusion MRI (dMRI) scans before (PRE) and after (POST) QPS.

The study comprised three days of experiments (Fig. 1). On Day 1, task functional MRI (fMRI) data were collected to localize the M1 responsible for the right first dorsal interosseous (FDI) muscle. Structural MRI data were collected on the same day. On Days 2 and 3, participants underwent either QPS5 or QPS50. Resting-state fMRI (rsfMRI) and dMRI data were obtained before and after QPS to assess functional and microstructural changes, respectively. Experiments on Days 2 and 3 were performed in the afternoon, from 1 pm to 3 pm, and were separated by more than a week to avoid any cumulative effects. QPS5 and QPS50 protocols were randomly assigned to Days 2 and 3, and the order was counterbalanced across participants.

### 2.1 Participants

Sixteen healthy adult volunteers (eight males; age range 20–25 years, mean 22.7 years, standard deviation [SD] 1.4 years) participated in this study. All participants were right-handed according to the Edinburgh handedness inventory (mean score 95.05, SD 9.8), and reported no history of neurological or psychiatric disorders. The experiments were approved by the Institutional Ethics and Safety Committees of the National Institute of Information and Communications Technology and were performed in accordance with the Declaration of Helsinki. Informed consent was obtained from all participants after fully explaining the study protocols and objectives.

### 2.2 Qps

Participants underwent QPS5 and QPS50 over the left M1, targeting the right FDI muscle, using the same procedure described in a previous study (Kimura et al., 2022). In brief, the motor hotspot for the right FDI muscle was first determined. The coil orientation was then defined for each participant to evoke the largest MEPs, with the coil handle pointing posteriorly and approximately 45 degrees away from the midline (Hamada et al., 2008;Kimura et al., 2021,^2022^). This orientation was consistently applied throughout the study. From this hotspot, the active motor threshold (AMT) was defined (evoking MEPs larger than 100μV in 5 out of 10 trials with mild contraction of the right FDI muscle). The QPS was then applied with a burst of four monophasic pulses every 5 s for 30 min, and the stimulus intensity was set at 90% of the AMT. The inter-pulse interval for QPS5 was 5 ms, whereas that for QPS50 was 50 ms. These procedures were performed using a DuoMAG MP-Quad (Deymed Diagnostic s.r.o., Hronov, Czech Republic) equipped with a butterfly-shaped 70-mm air-cooling coil (DuoMAG 70BF Air Cooled Coil; Deymed Diagnostic s.r.o., Hronov, Czech Republic), and MEPs were collected using a Brainsight surface electromyogram (Rogue Research Inc., Montreal, Canada). The position was recorded using Brainsight to quantitatively assess the stability of the coil position during QPS. The mean distance error of the coil position and the mean angle or twist error of the coil handle relative to the target position of the coil were calculated in each QPS session to evaluate the quality of applying QPS in each session.

### 2.3 Image acquisition

On Day 1, two sessions of task fMRI data during a finger-tapping task and structural MRI data were acquired using a Siemens PrismaFit 3T scanner equipped with a 32-channel array head coil (Siemens, Erlangen, Germany). On Days 2 and 3, rsfMRI and dMRI data before and after QPS were obtained using a Siemens Vida 3T scanner equipped with a 64-channel array head-neck coil (Siemens, Erlangen, Germany).

Two sessions of fMRI data were collected while participants tapped their right or left index fingers, alternated across blocks, as described in a previous study (Watanabe et al., 2014). These sessions were aimed to identify the left and right M1 responsible for the FDI muscles, which were subsequently used to calculate the FC (Watanabe et al., 2014) (seeSection 2.4.). Each session comprised six blocks of 20 s, with a 20 s inter-block interval, and lasted 4 min 20 s. In each block, participants were required to tap their left or right index fingers synchronized with the central red circle (radius 1 degree) flashing at 1 Hz. These fMRI data were collected with a gradient-echo echo-planar sequence, and the slices were collected in an interleaved order. The other acquisition parameters were as follows: flip angle = 60 degrees; voxel size = 2 × 2 × 2 mm, matrix size = 108 × 108 × 78, multiband acceleration factor = 6, phase-encoding direction = anterior-posterior, repetition time (TR) = 1000 ms, and echo time (TE) = 30 ms. A pair of B0 field maps in phase-encoding directions (i.e., anterior-posterior and posterior-anterior) were also obtained to correct susceptibility-induced distortion in fMRI data. These field maps were collected using a spin-echo echo-planar sequence with the following parameters: flip angle = 90 degrees, voxel size = 2 × 2 × 2 mm, matrix size = 108 × 108 × 78, multiband acceleration factor = 1, TR = 8330 ms, and TE = 63.40 ms.

T1-weighted (T1w) and T2-weighted (T2w) images were obtained for neuronavigation of the TMS coil during QPS on Days 2 and 3 and for spatial normalization and surface-based analysis of the MRI data. A T1w image was obtained with a magnetization-prepared rapid acquisition with a gradient echo sequence with the following parameters: flip angle = 8 degrees, voxel size = 0.8 × 0.8 × 0.8 mm, matrix size = 224 × 320 × 320, inversion time (TI) = 1000 ms, TR = 2500 ms, and TE = 2.18 ms. A T2w image was obtained with a sampling perfection with application-optimized contrast using different flip angle evolution sequences with the following parameters: voxel size = 0.8 × 0.8 × 0.8 mm, matrix size = 224 × 320 × 320, TR = 3200 ms, and TE = 564 ms. The total acquisition time for these two images was approximately 10 min.

On Days 2 and 3, rsfMRI data were collected to assess QPS-induced functional changes. These data were acquired using the same acquisition sequence and parameters as the task fMRI data on Day 1. During the scans, participants were required to fixate their gaze at a black cross displayed at the center of a screen for 10 min without engaging in any particular thoughts. As with the task fMRI on Day 1, a pair of B0 field maps in both phase-encoding directions was obtained with the following parameters: flip angle = 90 degrees, voxel size = 2 × 2 × 2 mm, matrix size = 108 × 108 × 78, multiband acceleration factor = 1, TR = 6240 ms, and TE = 44.00 ms. After each scan, participants were instructed to self-evaluate their sleepiness during the scan using the Stanford Sleepiness Scale (SSS;Hoddes et al., 1972).

dMRI data were acquired to assess QPS-induced microstructural changes. These data were obtained using a two-dimensional single-shot spin-echo echo-planar sequence with the following parameters: voxel size = 2 × 2 × 2 mm, matrix size = 106 × 106 × 75, iPAT reduction factor = 2, multiband acceleration factor = 3, b = 1000 s/mm^2^, number of directions = 30, TR = 5000 ms, and TE = 71 ms. Two sets of dMRI data were collected before and after QPS: one with the phase-encoding direction anterior-posterior and the other with the reversed phase-encoding direction (posterior-anterior). Three non-diffusion-weighted images for each dMRI data were also obtained in every 10 diffusion-weighted images using the same acquisition parameters as the diffusion-weighted images. The total acquisition time for dMRI was approximately 7 min in each session.

### 2.4 Image analysis

Unless otherwise specified, the default settings of the preprocessing pipelines of the Human Connectome Project (HCP pipelines 4.3.0,https://github.com/Washington-University/HCPpipelines) were utilized for preprocessing MRI data. The methods are detailed byGlasser et al. (2013). These pipelines utilize the FMRIB software library (FSL 6.0.5,https://fsl.fmrib.ox.ac.uk/fsl), FreeSurfer (Version 6.0.1,https://surfer.nmr.mgh.harvard.edu/;Dale et al., 1999;Fischl et al., 1999), and Connectome Workbench (Version 1.5.0,https://www.humanconnectome.org/software/connectome-workbench) to optimally preprocess MRI data obtained in an HCP-like fashion and to create the data in the left or right cerebral cortex and subcortical regions with the Connectivity Informatics Technology Initiative (CIFTI) format (seeSupplementary Methodsfor the details of preprocessing steps).

After preprocessing, the left M1 responsible for the right FDI muscle and its contralateral were defined from task fMRI data. A general linear model (GLM) analysis of the task fMRI data was performed using*TaskfMRIAnalysis.sh*in the HCP pipelines to define the left M1 responsible for the right index finger. This script performs surface-based analysis using the FEAT tools of FSL. First, the data were high-pass filtered with a cut-off period of 160 s, and the event timings for tapping the right or left index finger were created with a boxcar design in each session, with the contrast tapping the right index finger larger than the left one (RIGHT > LEFT). After applying a first-level GLM analysis, a second-level GLM analysis was performed across sessions for each participant. Finally, a permutation analysis of linear models (PALM) application of FSL was used to perform a one-sample t-test towards the RIGHT > LEFT contrast across participants on the fsaverage 32k surface for cerebral cortex and in the MNI standard space for subcortical regions.

Seed-based correlation analyses were performed on the rsfMRI data to quantify QPS-induced functional changes in the stimulated and unstimulated regions. FC was used for this purpose because QPS bidirectionally modulated this measure between stimulated and unstimulated regions (Watanabe et al., 2014). The seed was defined as a 5-mm radius of the peak location identified by the RIGHT > LEFT contrast of the group-level GLM analysis in task fMRI (the stimulated location) following a previous study (Watanabe et al., 2014). Pearson’s correlation coefficient was calculated between the time series of the seed and that of each vertex or voxel in the whole brain. These coefficients were transformed using Fisher’s z-transformation to conform to normal distributions.

As a previous study has reported that the change in the FC between the left and right M1 correlated with the magnitude of the after-effect of QPS over left M1 (Watanabe et al., 2014), Fisher’s z-transformed Pearson’s correlation was further calculated between the time series of the left and right M1. The right M1 was defined as a 5-mm radius of the peak location in the LEFT > RIGHT contrast. The functional change after QPS was measured by subtracting the FC of the bilateral M1 after QPS from that before QPS.

Fractional anisotropy (FA) and mean diffusivity (MD) values were calculated from dMRI data using a command*dtifit*in FSL. Subsequently, data from the cerebral cortex were resampled to fsaverage 32k surface, and the subcortical regions were warped to the MNI standard space using*NoddiSurfaceMapping*(https://github.com/RIKEN-BCIL/NoddiSurfaceMapping), as previously described in detail byFukutomi et al. (2018). In brief, data within the cerebral cortex were projected onto the mid-thickness surface and resampled to fsaverage 32k surface using MSMAll (see*Mutlimodal Surface Matching*in theSupplementary Methods), whereas those in the subcortical regions were warped to the MNI standard space. All these data were spatially smoothed with a Gaussian kernel of 2 mm full-width half maximum.

The tract-based spatial statistics (TBSS) tool of FSL was applied to assess changes in the microstructural properties of the white matter (Smith et al., 2006). This method generates statistics for group-level voxel-wise analysis in the white matter, as detailed in a previous study (Kimura et al., 2021). In brief, the FA map for each individual was non-linearly registered to the MNI standard space, the main white matter structure (skeleton) was created from the spatially normalized FA maps of all participants, and FA and MD values were projected onto this skeleton.

### 2.5 Statistical analysis

The PALM application was used to test the statistical significance of functional (i.e., the FC of the left M1) or microstructural (i.e., FA and MD values) changes after QPS5 or QPS50 and the difference between changes after QPS5 and QPS50. Specifically, we performed 2000 sign-flipping permutation tests using the tail approximation method (Winkler et al., 2016) combined with threshold-free cluster enhancement (Smith & Nichols, 2009).*P*-values were corrected for family-wise error both within each dataset and across comparisons (e.g., pre-QPS5 vs. post-QPS5 or pre-QPS50 vs. post-QPS50) and modalities (e.g., FA or MD value) (Alberton et al., 2020). The correction was performed using the Westfall-Young method (Westfall & Stanley Young, 1993). The data from the left and right cerebral cortex and subcortical regions were stored in the same CIFTI file, while the surface (the left and right cerebral cortex) and volumetric (subcortical regions) data require different options for threshold-free cluster enhancement. Thus, we first separated the CIFTI files into three structures: the left and right cerebral cortex and subcortical regions. Next, we applied PALM to each of these separated structures and further corrected the*P*-values with the Dunn–Šidák correction over the separated structures.

The Bayes factor was additionally calculated for each comparison of microstructural property to assess the degree of support for the null hypothesis in each statistical test for non-significantly different areas. The null hypothesis (H_0_) was defined as the effect size being zero, while the alternative hypothesis (H_1_) as not being zero. The Jeffreys-Zellner-Siow prior was used for the prior distribution (Rouder et al., 2009) with the default scale parameter of$\sqrt{2}$/2. The Bayes factor was calculated using*BayesFactor*ver. 0.9.2 in*R*ver. 4.1.2.

PALM was additionally utilized to examine the correlation between functional change (the FC change between the bilateral M1) and the change in FA or MD values after QPS5 and QPS50. The default permutation tests (not sign-flipping permutation tests) were conducted with the same settings and correction methods as described above.

To evaluate the degree of impact of possible confounders to the neuroimaging results, we compared the imaging quality of dMRI or rsfMRI and sleepiness during rsfMRI scans before and after QPS and changes in these values between after QPS5 and QPS50. Namely, the following measures were compared: mean framewise displacement (FD;Power et al., 2014) and SSS for rsfMRI data, while mean absolute and relative head motions (Bastiani et al., 2019;Oldham et al., 2020) for dMRI data. The mean absolute and relative head motions and mean FD were compared using a two-tailed paired t-test. As SSS is an ordinary scale, it was compared using a two-tailed Wilcoxon signed-rank test. These analyses were performed using the JASP (ver. 0.16.0 for Windows,https://jasp-stats.org/), and statistical significance was set at*P*< 0.05.

## 3 Results

**Fig. 2.**
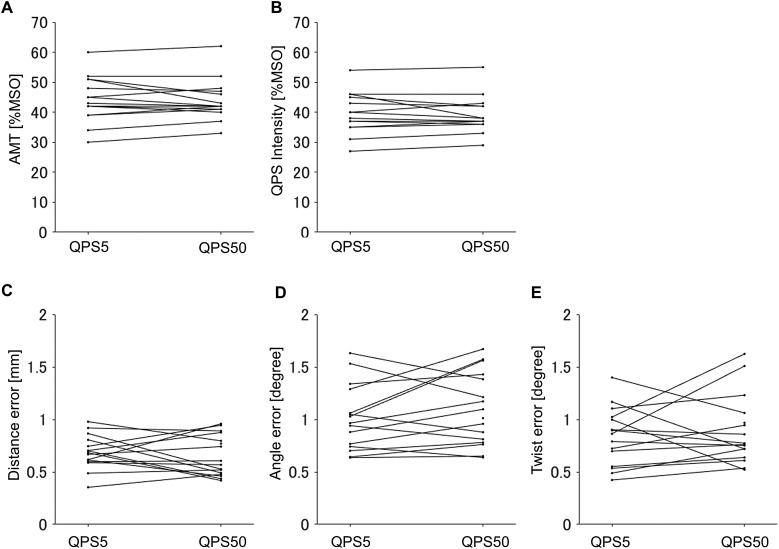
Comparison of baseline neurophysiological characteristics (A and B) and the quality of applying QPS (C–E) between QPS5 and QPS50 conditions. The active motor threshold (AMT) and stimulus intensity for applying QPS (QPS intensity) are shown in (A) and (B), respectively. The vertical axes represent the percentage of maximum stimulator output (%MSO). The mean distance error (mm; C), the mean angle error (degree; D), and the mean twist error (degree; E) in each session are also shown.

All 16 healthy adults completed the 3-day experiment without experiencing any adverse events. The two sessions of the task fMRI and the outputs from a neuronavigation system in one session of the QPS5 condition were not obtained owing to technical issues. Paired t-tests indicated no significant differences in AMT (*t*= 0.40,*P*= 0.70;Fig. 2A) or stimulus intensity for QPS (*t*= 0.48,*P*= 0.64;Fig. 2B) between the QPS5 and QPS50 conditions. Similarly, no significant differences were observed in the quality of the QPS sessions (mean distance error,*t*= 0.77,*P*= 0.46,Fig. 2C; mean angle error,*t = *-1.37,*P = *0.19,Fig. 2D; mean twist error,*t = *-0.56,*P*= 0.59,Fig. 2E).

### 3.1 Changes in the microstructural indices after QPS

**Fig. 3.**
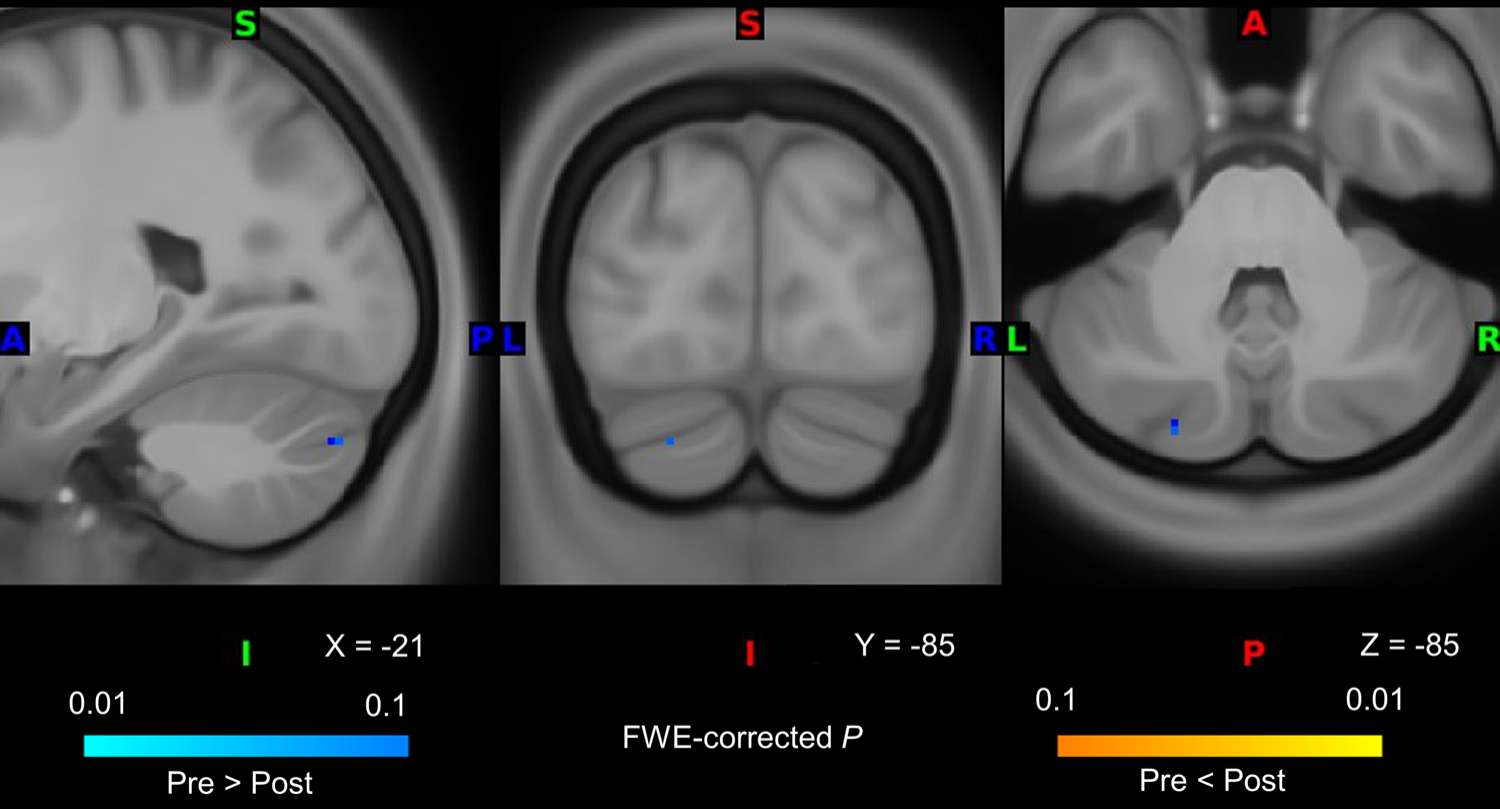
Differences in mean diffusivity (MD) between pre- and post-QPS5. Areas in blue show that MD value significantly decreased after QPS5, whereas areas in yellow indicate that it significantly increased after QPS5 (not found). Axial and coronal slices are shown in accordance with neurological conventions (the left side of the image is the left side of the brain). The value was thresholded with family-wise-error-corrected*P*< 0.1 only for visualization purposes.

**Fig. 4.**
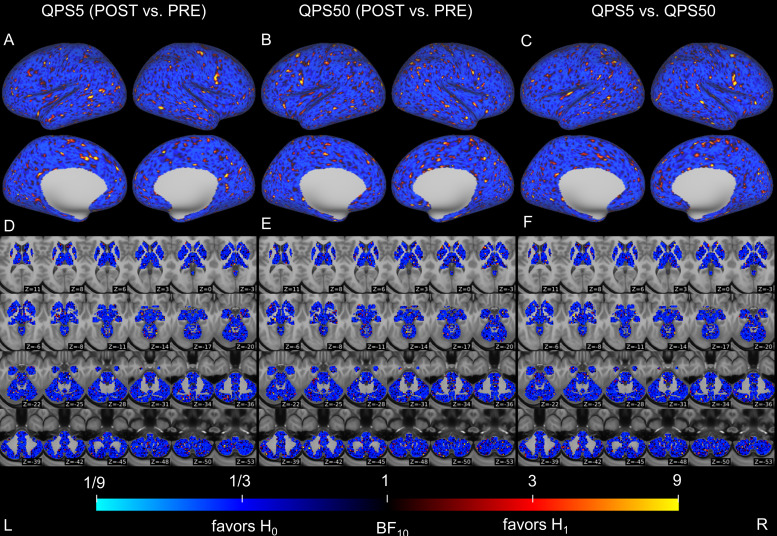
Differences in fractional anisotropy (FA) values of the cerebral cortex and subcortical regions between pre- and post-QPS in the QPS5 (A and D) and QPS50 (B and E) conditions, as well as in changes in FA values across conditions (C and F). The upper panels (A–C) show the result of surface-based analysis on the cerebral cortex, whereas the lower panels (D–F) show the result of voxel-based analysis in the subcortical regions. Areas in blue indicate that the Bayes factor is less than 1 (relatively supporting the hypothesis that the effect size was zero [H_0_]) when comparing FA values before and after QPS or changes in the FA values between conditions. In contrast, areas in yellow show higher than 1 (relatively supporting the hypothesis that the effect size was not zero [H_1_]). Axial slices in (D–F) are shown in accordance with neurological conventions (the left side of the image represents the left side of the brain) and are displayed in the MNI coordinates from z = 11 (top left) to z = -53 (bottom right).

**Fig. 5.**
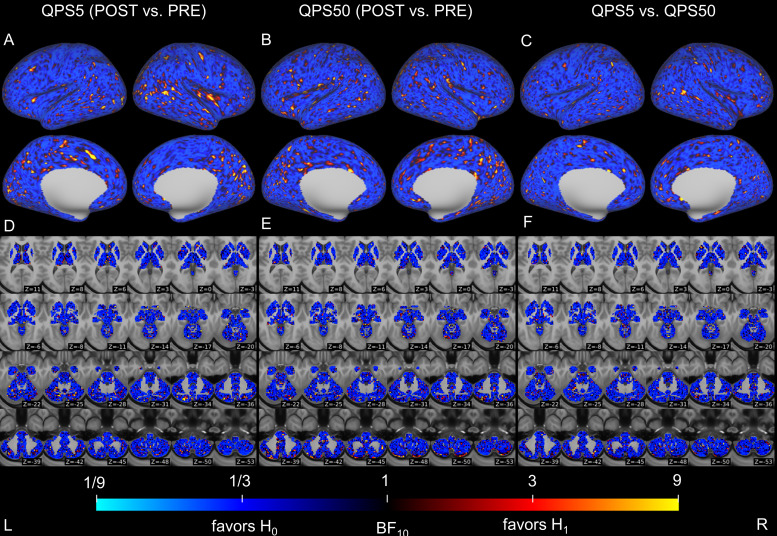
Differences in mean diffusivity (MD) values of the cerebral cortex and subcortical regions between pre- and post-QPS in the QPS5 (A and D) and QPS50 (B and E) conditions as well as in changes in MD values across conditions (C and F). The upper panels (A–C) show the result of surface-based analysis on the cerebral cortex, whereas the lower panels (D–F) show the result of voxel-based analysis in the subcortical regions. Areas in blue indicate that the Bayes factor is less than 1 (relatively supports the hypothesis that the difference was zero [H_0_]) when comparing the MD values before and after QPS or the change in MD values across conditions. In contrast, areas in yellow show higher than 1 (relatively supporting the hypothesis that the effect size was not zero [H_1_]). Axial slices in (D–F) are shown in accordance with neurological conventions (the left side of the image is the left side of the brain) and are displayed in the MNI coordinates from z = 11 (top left) to z = -53 (bottom right).

We evaluated alterations in microstructural properties within the gray matter of the cerebral cortex and subcortical regions following QPS5 or QPS50 by comparing FA and MD values before and after QPS5 or QPS50. In the cerebral cortex, we observed no significant changes after QPS5 or QPS50 in FA and MD values. By contrast, a significant decrease in MD values was observed in the left cerebellum following QPS5 (Fig. 3), although its significance disappeared after regressing out mean absolute and relative head motions from the FA or MD values (see*Sensitivity analyses*inSupplementary Resultsfor details). Except for this change, no significant alterations were observed in subcortical regions for FA and MD values. Moreover, no significant difference was observed in the changes in FA and MD values between the QPS5 and QPS50 conditions. For each comparison, the Bayes factor relatively supported the hypothesis that the effect size is zero (H_0_) over the alternative one (H_1_) in most regions (FA:Fig. 4; and MD:Fig. 5).

Subsequently, we assessed alterations in the microstructural properties of the white matter following QPS5 and QPS50. TBSS analysis revealed no significant changes after QPS5 or QPS50 in FA and MD values. No significant differences were observed in the changes in FA and MD values in the white matter between QPS5 and QPS50 conditions. We repeated the same analyses after regressing out mean absolute and relative head motions from the FA or MD values, and confirmed that the conclusions drawn from the results remained the same (see*Sensitivity analyses*inSupplementary Resultsfor details). For each comparison, the Bayes factor relatively supported the hypothesis that the effect size is zero (H_0_) over the alternative one (H_1_) in most regions (FA:Supplementary Fig. 7; and MD:Supplementary Fig. 8).

### 3.2 Changes in the FC after QPS and their relationship with changes in the microstructural indices

**Fig. 6.**
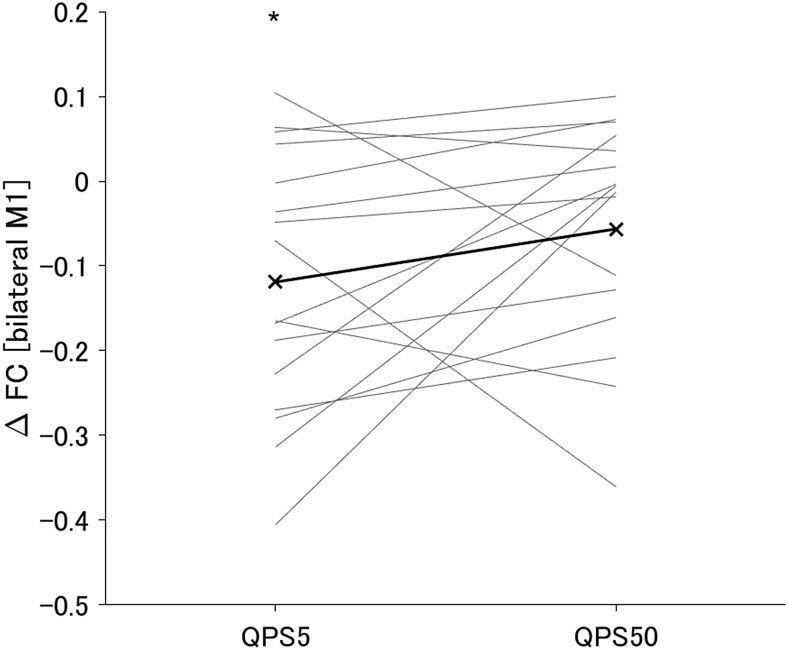
Changes in the functional connectivity (FC) between the left and right M1 after QPS5 or QPS50. Thick black crosses indicate the mean FC change across participants. The y-axis indicates the change in the FC after QPS. *shows that the change was statistically significant (*P < *0.05).

We first examined the functional changes after QPS5 or QPS50 by assessing the change in the FC of the stimulated region (the left M1). After QPS5, the FC of the left M1 was significantly decreased in the right M1 and primary somatosensory cortex, while it significantly increased in the bilateral cerebellum (Supplementary Fig. 9). By contrast, following QPS50, no significant change was detected in the FC. We also found no significant difference between the changes in FC of the left M1 after QPS5 and QPS50. We further compared the change in FC specifically between the left and right M1 because a previous study suggested that this change can reflect the degree of the after-effect of QPS on the stimulated region (Watanabe et al., 2014). We observed large inter-individual variability in the change of the FC between the left and right M1 following both QPS5 and QPS50 (Fig. 6). Following QPS5, the FC between the bilateral M1 was significantly decreased after QPS5 (*t*= -3.08,*P*= 0.023), whereas the FC was not significantly modulated following QPS50 (*t*= -1.71,*P*= 0.32) (Fig. 6). No significant differences were observed between QPS5 and QPS50 conditions regarding the change in the FC between these two regions after QPS (*t*= -1.43,*P = *0.52). Subsequently, we examined the correlation between changes in FA or MD values and the change in FC between the bilateral M1 following QPS. Regression analysis revealed no significant correlations between changes in FA or MD values and those in FC between the left and right M1 after QPS5 or QPS50. We repeated the same analyses as above after regressing out mean FD or mean FD and SSS, and confirmed that the conclusions drawn from both sets of the results remained the same (see*Sensitivity analyses*inSupplementary Resultsfor details).

As QPS5 significantly decreased the FC between the left and right M1, we additionally investigated the FA or MD changes after QPS5 in the corpus callosum (CC) connecting the left and right M1 (see*Tractography*inSupplementary Methods). The CC was specifically investigated because a previous study reported changes in FA in the CC after modulating the FC between the bilateral primary somatosensory/motor cortex (Sampaio-Baptista et al., 2021). As a result, we observed no significant change in the FA (*t*= 0.99,*P = *0.34,Supplementary Fig. 10A) or MD (*t*= -1.35,*P*= 0.20,Supplementary Fig. 10B) value in the CC after QPS5. We did not observe a significant correlation between the mean decrease in the MD value and the mean change in FC of the left M1 within the cluster of the left cerebellum whose MD values significantly decreased after QPS5 (rho = -0.23,*P*= 0.40).

### 3.3 Potential confounders within and across sessions

**Fig. 7.**
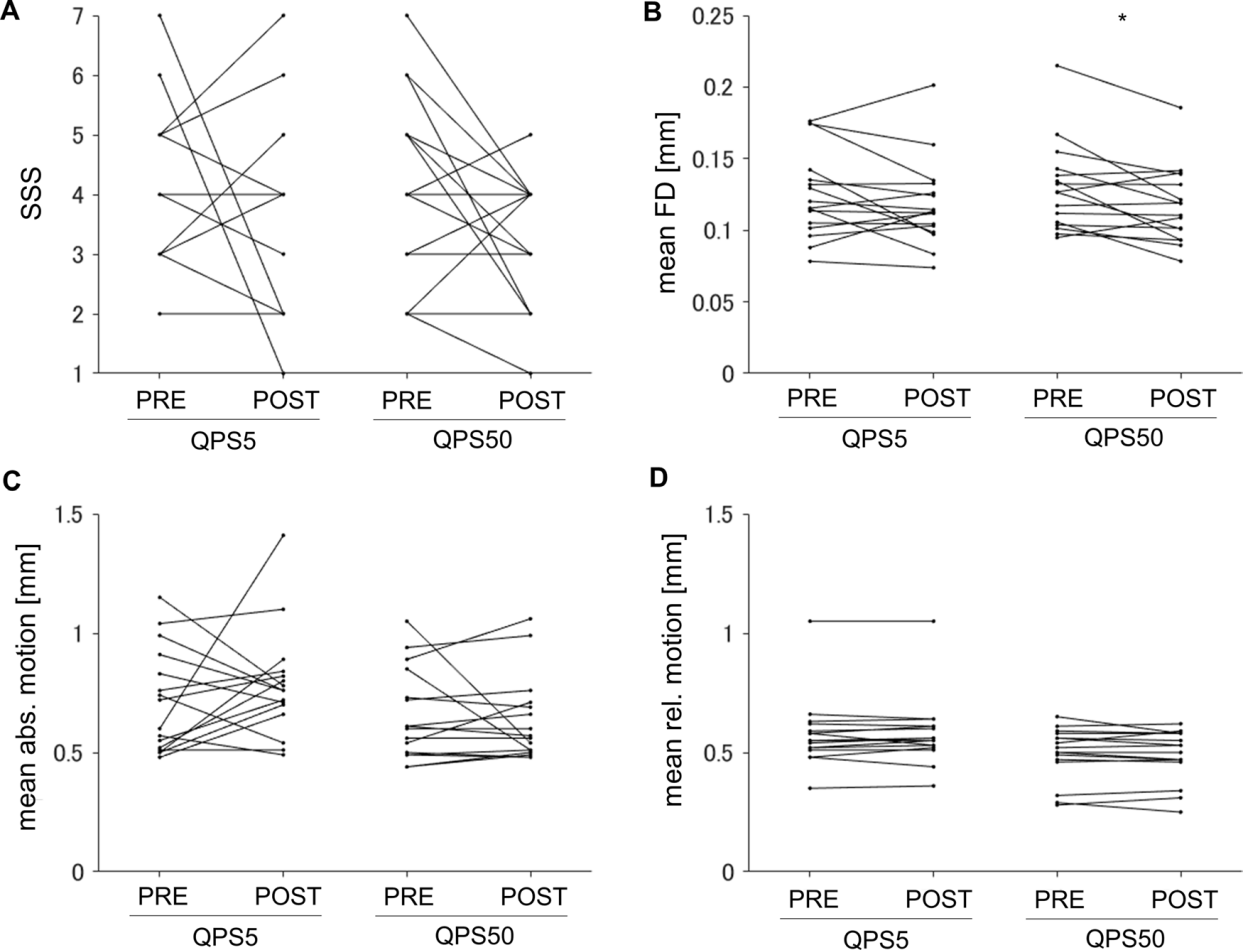
(A–D) Comparisons of the sleepiness during resting-state fMRI (rsfMRI) and imaging qualities of rsfMRI and diffusion MRI (dMRI) measured pre- and post-QPS in the QPS5 and QPS50 conditions. (A) and (B) show the Stanford Sleepiness Scale (SSS) and mean framewise displacement (FD; mm) during resting-state fMRI scan, respectively, whereas (C) and (D) illustrate mean absolute and relative head motions (mm) during dMRI scan. * indicates that the comparison was significantly different according to the paired t-test.

To identify confounding factors affecting changes following QPS5 or QPS50 and differences in these changes between the two conditions, we compared quantitative measures of sleepiness during rsfMRI scans and image quality of rsfMRI and dMRI before and after QPS, as well as changes in these measures between the two conditions. As for rsfMRI, Wilcoxon signed-rank tests revealed no significant changes in the SSS scores after QPS5 or QPS50 (QPS5,*W*= 65.50,*P*= 0.41; QPS50,*W*= 63.00,*P*= 0.61;Fig. 7A). No significant change in the mean FD was observed after QPS5 (*t*= 1.24,*P*= 0.23,Fig. 7B); however, a significant decrease was observed after QPS50 (*t = *2.69,*P*= 0.017,Fig. 7B). Regarding dMRI image quality, we observed no significant changes after QPS5 (mean absolute motion,*t*= -0.99,*P*= 0.34,Fig. 7C; mean relative motion,*t = *-0.52,*P*= 0.61,Fig. 7D) or QPS50 (mean absolute motion,*t*= 0.51,*P*= 0.62,Fig. 7C; mean relative motion,*t*= 0.78,*P*= 0.45,Fig. 7D). No significant difference was observed between QPS5 and QPS50 conditions in the change of any of these measures within the sessions (SSS,*W*= 49.50,*P*= 0.80,Fig. 7A; mean FD,*t = *0.99,*P*= 0.34,Fig. 7B; mean absolute motion in dMRI,*t = *1.23,*P*= 0.24,Fig. 7C; mean relative motion in dMRI,*t = *0.83,*P = *0.42,Fig. 7D).

## 4 Discussion

In this study, we investigated the immediate effect of QPS5 or QPS50 over the left M1 on the microstructural properties in the whole human brain. Our findings revealed no significant alterations in FA or MD values following QPS5 or QPS50, except for a significant decrease in MD values in the left cerebellum after QPS5. No significant correlations were observed between these microstructural changes and changes in FC between the bilateral M1, which may reflect the functional changes on the stimulated region after QPS over the left M1, in either the QPS5 or QPS50 condition.

### 4.1 No significant change in the microstructural property of the cerebral cortex after QPS

No significant changes in FA or MD values were observed in the cerebral cortex following QPS5 or QPS50. Additionally, no significant correlation was observed between alterations in FA or MD values and QPS-induced functional changes. Given the sensitivity of FA and MD values as biomarkers for detecting changes in the microstructural properties of neurons (Basser et al., 1994;Zhong et al., 1993) and glial cells (Debacker et al., 2020;Sagi et al., 2012;Yi et al., 2020) in the brain, our results suggest that the excitatory or inhibitory QPS protocol did not immediately modulate microstructural properties. Our results align with those of previous studies showing no significant changes in microstructural properties following the excitatory protocol of conventional rTMS in the human brain (Niehaus et al., 2000). In contrast, the previous studies have shown inconsistent results regarding the inhibitory protocol of conventional rTMS in humans: one study showed that this protocol decreased the MD value on the stimulated region (Mottaghy et al., 2003), which was not replicated in another study (Duning et al., 2004), while the other study reported an increase in MD value in both the stimulated and unstimulated regions (Abe et al., 2014). These inconsistencies, even within studies using the same protocol of rTMS, may stem from large inter-individual variability in the degree of plasticity in conventional rTMS (Maeda et al., 2000;Prei et al., 2023). Considering the lack of effects in the current study using QPS, which induces brain activity changes on the stimulated region robustly across participants (Kimura et al., 2022;Nakamura et al., 2016;Tiksnadi et al., 2020), we speculate that the inhibitory protocol of rTMS does not immediately alter the microstructural property after the stimulation.

Several animal rTMS studies have shown that a single session of rTMS can alter the microstructure several hours after stimulation. For example, the excitatory protocol of conventional rTMS increased the dendritic spine size of the pyramidal neurons (Vlachos et al., 2012), which can decrease the MD value, and morphometrically altered the inhibitory interneurons (Lenz et al., 2016) 2 to 4 hrs after the stimulation. In contrast, the inhibitory protocol of conventional rTMS has been shown to decrease the number of calbindin inhibitory interneurons 1 hr after the stimulation (Benali et al., 2011), which can increase the MD value. Furthermore, both the excitatory and inhibitory protocols of conventional rTMS have been reported to modulate the protein levels within the astrocytes 5 hrs after the stimulation (Clarke et al., 2021). These inconsistencies may be at least partly attributed to the difference in the timing of microstructural property evaluations following rTMS. Future studies will be required to clarify whether the microstructural property changes occur several hours after QPS.

### 4.2 Inconsistencies with the results on behavioral training

Previous studies have shown that the MD value in the gray matter decreased immediately following a behavioral training, such as spatial-memory task (Sagi et al., 2012) and piano training (Tavor et al., 2020). The discrepancies between the current study and these previous studies may be explained by several differences between QPS and behavioral training. First, QPS stimulates the brain extrinsically, while behavioral training induces intrinsic brain activation. A previous study revealed that the degree of plasticity after rTMS depends on the shape of the TMS pulse (Goetz et al., 2016), and microstructural plasticity may be induced by TMS pulses resembling natural neural activation. Second, the brain regions affected during QPS are more restricted than those during these behavioral training. A previous study showed that the brain activity affected during QPS5 over the left M1 was restricted to the ipsilateral premotor cortex and M1 (Groiss et al., 2013), whereas more widespread brain regions, such as bilateral fronto-parietal and sensory-motor networks, are activated during spatial-memory tasks (Casey et al., 1998) and piano training (Olszewska et al., 2024). Third, even if QPS is applied to one of the regions activated during behavioral training, the areas activated by QPS would be more restricted than those activated by behavioral training when compared within the same area. While behavioral training activates neurons in both superficial and deep layers of the cortex (Yu et al., 2022), a subthreshold (i.e., lower than RMT) TMS pulse has been proposed to mainly activate the axon terminal of superficial pyramidal neurons within the cortex (Siebner et al., 2022). Broader activation of neurons across layers would increase the possibility of activating astrocytes within the cortex, thereby increasing its size (Johansen-Berg et al., 2012). Therefore, we speculate that extrinsic repetitive stimulation of a single brain region by TMS may be insufficient to evoke immediate microstructural alterations in the human brain.

### 4.3 The decrease in the MD values of the left cerebellum after QPS5

The MD value in the left cerebellum decreased after QPS5, with no significant difference in the change between the QPS5 and QPS50 conditions. A recent study reported that the gray matter volume of the cerebellum decreased following the inhibitory protocol of rTMS over the anterior temporal lobe (Jung & Lambon Ralph, 2021). Therefore, QPS5 may also induce plasticity in the left cerebellum, leading to microstructural alterations in the same region. However, cautions are warranted in interpreting this result owing to the absence of control conditions in this study, such as a sham condition or QPS over a different region. Moreover, the left cerebellum is not structurally connected to the left M1 (i.e., the stimulated region) via cortico-ponto-cerebellar pathways (Kamali et al., 2010), which means that the left cerebellum is less likely to be affected by QPS5 over the left M1. Additionally, voxel-based analysis with dMRI, as opposed to the surface-based analysis used in the cerebral cortex, may be prone to registration errors (Jones et al., 2005). Future animal studies or MRI studies with high spatial resolutions are required to further validate this finding.

### 4.4 No significant change in the microstructural property of the white matter after QPS

The FA or MD values in the white matter did not change following QPS5 or QPS50. This result is consistent with that of a prior study that showed no significant immediate modulation of the white matter volume following continuous theta-burst stimulation, an inhibitory patterned rTMS protocol, over the temporal area (Jung & Lambon Ralph, 2021). By contrast, several studies have reported microstructural alterations in the white matter 24 hr after neurofeedback training (Sampaio-Baptista et al., 2021) and cortico-cortico paired associative stimulation (Lazari et al., 2022). Furthermore, repetitive optogenetic stimulation over the neurons in the premortor cortex induced oligodendrogeneis in the white matter underneath the stimulated region a few hours after the stimulation in mice (Gibson et al., 2014). These differences may be attributed to the differences in the time evaluated after intervention. Given that the after-effect of QPS lasts for more than several hours (Hamada et al., 2008), microstructural changes in the white matter may also be observed several hours after QPS.

### 4.5 FC between the bilateral M1 decreased after QPS5 but was not significantly modulated after QPS50

In the current study, the FC between the left and right M1 decreased following QPS5, but was not significantly modulated after QPS50, showing huge inter-individual variability. These results partially contradict those of a previous study that reported a decrease in FC between these two regions after QPS5 and an increase after QPS50 (Watanabe et al., 2014). These differences between the studies could be explained by the timing of measurements after QPS: our study assessed FC immediately after QPS, whereasWatanabe et al. (2014)evaluated FC 30 min after QPS. Considering that bidirectional changes in the MEP amplitude after QPS over the left M1 were clearly observed 30 min after QPS (Kimura et al., 2022), alterations in FC between the bilateral M1 may also be observed more clearly 30 min after QPS than immediately after QPS.

### 4.6 Limitations

This study has some limitations. First, we did not compare the changes in microstructural or functional properties after QPS with those observed in control conditions, such as those after sham rTMS or QPS applied to a different location. Therefore, the significant modulations observed after QPS may not be directly related to the application of rTMS over the left M1, potentially introducing confounders such as registration errors in image analysis.

The second limitation lies in the limited sample size in this study. While the sample size in our study (16 participants) is comparable to those in previous studies demonstrating significant alterations in microstructural properties after rTMS (12 participants inAbe et al. (2014)and 8 inMottaghy et al. (2003)), a recent study evaluated microstructural alterations after neural interventions using a larger sample size (28 participants inSampaio-Baptista et al. (2021)). Future large-scale sham-controlled MRI studies are crucial to validate our results.

The third limitation is that we did not directly assess the cortical excitability in the stimulated region after QPS. This is because we wanted to minimize the time between the end of QPS and the start of the MRI scan. Instead, using the same QPS procedure in the current study, we previously confirmed that the modulation of MEP by QPS persisted well beyond the time period of the MRI scan in the current study (up to 90 min after QPS) (Kimura et al., 2022). Moreover, the facilitatory and inhibitory effects of QPS5 and QPS50, respectively, were robust across participants: QPS5 over the left M1 increased MEP in 12 out of 12 participants, while QPS50 decreased MEP in 10 out of 12. Nevertheless, we cannot fully rule out the possibility that the negative results on microstructural changes in the cerebral cortex are simply because QPS did not robustly induce plasticity in the stimulated region in this study.

### 4.7 Future directions

One of the future directions for this study would be to assess the microstructural changes using different modalities. We evaluated microstructural properties using metrics derived from dMRI. Although these metrics are useful in exploring changes in the microstructural properties of the human brain (Basser et al., 1994;Debacker et al., 2020;Komaki et al., 2020;Sagi et al., 2012), a previous study detected microstructural alterations after neural interventions using magnetization transfer imaging (Lazari et al., 2022), which is a sensitive biomarker for myelin. Given that the microstructural metrics derived from different modalities can capture different aspects of microstructural characteristics (Lipp et al., 2019), microstructural indices obtained with neuroimaging modalities other than dMRI may detect changes in microstructural properties after QPS.

Another interesting future direction is to assess the influence of rTMS on astrocytes or microglial cells. Several animal studies have suggested that rTMS can modulate the activity of glial cells (Cullen & Young, 2016). Given that the functional properties of glial cells can be noninvasively evaluated in the human brain using positron emission tomography (Maeda et al., 2004), it would be tempting to investigate the correlations between functional changes in glial cells and microstructural changes in the human brain following QPS.

## 5 Conclusion

No significant microstructural changes were observed in the human brain immediately after QPS5 or QPS50, except for the decrease in MD values in the left cerebellum after QPS5. These results may be interpreted as extrinsic repetitive brain activation of a single brain area is insufficient to change the microstructure of the human brain immediately after the intervention. However, this interpretation warrants caution because we did not evaluate the changes in brain activity in the stimulated region after QPS in the present study. Future large-scale sham-controlled studies will examine microstructural changes at different time points after the intervention using multiple imaging modalities.

## Supplementary Material

Supplementary Material

## Data Availability

The data used in this study will be available by the corresponding authors on reasonable request, and all codes are available on GitHub (https://github.com/ikko-kimura/QPS-MRI).
