# Supplementary material for: Immediate effect of quadri-pulse stimulation on human brain microstructures and functions
Source: Imaging Neurosci (Camb). 2024 Aug 12;2:imag-2-00264. doi: 10.1162/imag_a_00264 (PMC12290712; doi:10.1162/imag_a_00264)
Supplement: Supplementary Material [file imag_a_00264-supp.pdf]

Supplementary Methods

Preprocessing

Structural MRI data

Structural MRI data obtained on Day 1 (i.e., T1w and T2w images) were preprocessed using "PreFreeSurferPipeline.sh," "FreeSurferPipeline.sh," and "PostFreeSurferPipeline.sh" in the HCP pipelines. Gradient nonlinearity correction was not applied because all participants' heads were placed in the conventional location (i.e., the isocenter) of the MR scanner. First, both T1w and T2w images were transformed to the AC-PC space by rigid-body transformation to the Montreal Neurological Institute (MNI) standard space and brain extracted. A T2w image was co-registered to a T1w image, and the bias field was estimated from both the T1w and T2w images. After the bias field correction with this estimated bias field, these images were registered to the MNI standard space, and a modified version of the recon-all command was applied in FreeSurfer to preprocess MRI data with sub-millimeter spatial resolutions. This command generates the boundaries between the cerebrospinal fluid and gray matter (pial surface) and those between the gray and white matter (white surface) and extracts the subcortical structure. The midpoints between the pial and white surfaces were further calculated to create the mid-thickness surface. This surface was utilized to project the fMRI or dMRI data for further surface-based analysis on the cerebral cortex. These surfaces were resampled to fsaverage 164k surface, and then to fsaverage 32k surface. Segmented subcortical

volumes were warped to the MNI standard space. Finally, T1w data were divided by T2w data to derive the myelin map (Glasser & Van Essen, 2011) to be projected onto the mid-thickness surface, which was utilized for multimodal surface matching in this study (See the *Multimodal surface matching* section below for more details).

Functional MRI data

Both task fMRI and rsfMRI data were first preprocessed using "fMRIVolumeProcessingPipeline.sh" and "fMRISurfaceProcessingPipeline.sh" in the HCP pipelines. First, image distortions caused by the inhomogeneity of the magnetic field were estimated using the corresponding B0 field maps. Then, with these estimated distortions, fMRI data were distortion corrected, and motion correction was applied with rigid-body transformation. The corrected fMRI data were co-registered to the T1w image in the AC-PC space with boundary-based registration and warped to the MNI standard space. The data in the cerebral cortex were projected onto the mid-thickness surface and resampled to the MNI standard space with spatial smoothing using a Gaussian kernel of two mm full-width half maximum (FWHM) along the surface. By contrast, the time series of the subcortical structures were extracted from the subcortical voxels defined from the structural MRI data using FreeSurfer. These voxels were warped to the MNI standard space and spatially smoothed with a Gaussian kernel of two mm FWHM.

To further denoise the time series of the rsfMRI data, the "hcp_fix" was applied to the HCP pipelines. This method is based on the FIX application of FSL, which performs single-run spatial independent component analysis towards fMRI data to remove the noise components from these time series (Griffanti et al., 2014). These components were manually re-inspected following the criteria described by Griffanti et al., (2017), and the noise components were regressed out from the time series. The mean framewise displacement (Power et al., 2014) was calculated for each session to quantitatively assess the quality of the rsfMRI data in each session.

Multimodal surface matching

To further fine-tune the resampling strategy of surface to fsaverage surface, "MSMAllPipeline.sh" was used in the HCP pipelines. This pipeline uses a multimodal surface matching tool (Robinson et al., 2014) of FSL customized for the HCP pipelines (MSMAll; Glasser et al., (2016); Robinson et al., (2018)), which utilizes multimodal features, such as the myelin map and the functional connectivity on each surface. Subsequent to MSMAll, the time-series fMRI data were resampled to the fsaverage 32k surface using the resampling strategy defined with this method.

Diffusion MRI data

To preprocess the dMRI data, "DiffPreprocPipeline.sh" was applied. First, non-diffusion-weighted images were intensity-normalized, and image distortions caused by the inhomogeneity of the magnetic field were estimated from these images. With these estimated distortions, the dMRI data were distortion corrected and then corrected for eddy current and subject motion. These corrected images were co-registered with the T1w image in AC-PC space using BBR. The quantitative measurements of the image qualities in each scan, namely absolute and relative head motions, were calculated using the EDDY QC tools in the FSL (Bastiani et al., 2019).

Tractography

To locate the corpus callosum (CC) area connecting between the left and right primary motor cortex (M1), tractography was performed using MRtrix3 (version 3.0.4 ; Tournier et al., (2019)). First, the basis functions for deconvolving the dMRI data were calculated using the "dhollander" algorithm (i.e., the basis function was estimated for gray matter, white matter, and cerebro-spinal fluid and for each b-value). These basis functions were used to calculate the fiber orientation density (FOD) on each voxel with multi-shell multi-tissue constrained spherical deconvolution. The tissue boundary between the white and gray matter for the seed of tractography was created with the outputs of preprocessed structural MRI data. One hundred million streamlines were generated with anatomically constrained tractography (maximum

length: 300 mm, cut off: 0.06) using "Second-order Integration over Fiber Orientation Distributions" algorithm. These streamlines were refined using "Spherical-deconvolution Informed Filtering of Tractograms 2" to reduce the overestimation of the number of streamlines. Subsequently, the streamlines connecting the left and right M1 were extracted. We used the same left and right M1 areas as utilized for the calculation of functional connectivity (i.e., 5-mm radius of the peak location from task fMRI). The CC was defined using the outputs from FreeSurfer, and the region-of-interest was defined as the area where the CC and the streamlines connecting the bilateral M1 intersected.

Supplementary Results

Sensitivity analyses

Microstructural changes after QPS

We repeated the same analysis after regressing out the potential confounders (i.e., mean absolute and relative head motions (Oldham et al., 2020)) from the FA and MD values. The analysis revealed that the FA or MD values were not significantly changed after QPS5 or QPS50 in any region. Additionally, no significant difference was observed in the change of FA or MD values between the QPS5 and QPS50 conditions. For each comparison, the Bayes factor relatively supported the hypothesis that the effect size is zero (H_0) over the alternative one (H_1) in most regions (FA: gray matter, Supplementary Figure 1, white matter, Supplementary Figure 2; and MD: gray matter, Supplementary Figure 3, white matter, Supplementary Figure 4).

Functional Connectivity changes after QPS

We repeated the same analysis after regressing out (1) mean framewise displacement (FD; Power et al., (2014)) or (2) mean FD and Stanford Sleepiness Scale (SSS) from the FC. Given that SSS is an ordinary variable, we binarized the score by four following the previous study (Kimura et al., 2022). After regressing out mean FD, the FC of the left M1 (Supplementary Figure 5B) was significantly decreased in the right M1 and primary somatosensory cortex (S1) and increased in the bilateral cerebellum after QPS5, while it was not significantly changed in

any region after QPS50. Additionally, we found no significant difference in the FC change of the left M1 between the QPS5 and QPS50 conditions. The FC between the left and right M1 (Supplementary Figure 6B) was decreased after QPS5 (t = -3.17, P = 0.018), while it was not significantly altered after QPS50 (t = -1.86, P = 0.25). The FC change was not significantly different between the QPS5 and QPS50 conditions (t = -1.40, P = 0.54). There was no significant correlation between the change in FA or MD values and that in the FC between the bilateral M1 in any region after QPS5 or QPS50.

After regressing out mean FD and SSS, the FC of the left M1 (Supplementary Figure 5C) was significantly decreased in the right M1 and S1 and increased in the bilateral cerebellum after QPS5, while it was not significantly changed in any region after QPS50. Additionally, we found no significant difference in the FC change of the left M1 between the QPS5 and QPS50 conditions. The FC between the left and right M1 (Supplementary Figure 6C) was decreased after QPS5 (t = -3.14, P = 0.021), while it was not significantly altered after QPS50 (t = -1.77, P = 0.29). The FC change was not significantly different between the QPS5 and QPS50 conditions (t = -1.44, P = 0.17). There was no significant correlation between the change in FA or MD values and the change in FC between the bilateral M1 in any region after QPS5 or QPS50. No significant correlation was also observed between these changes after regressing out mean absolute and relative head motions from the FA or MD values.

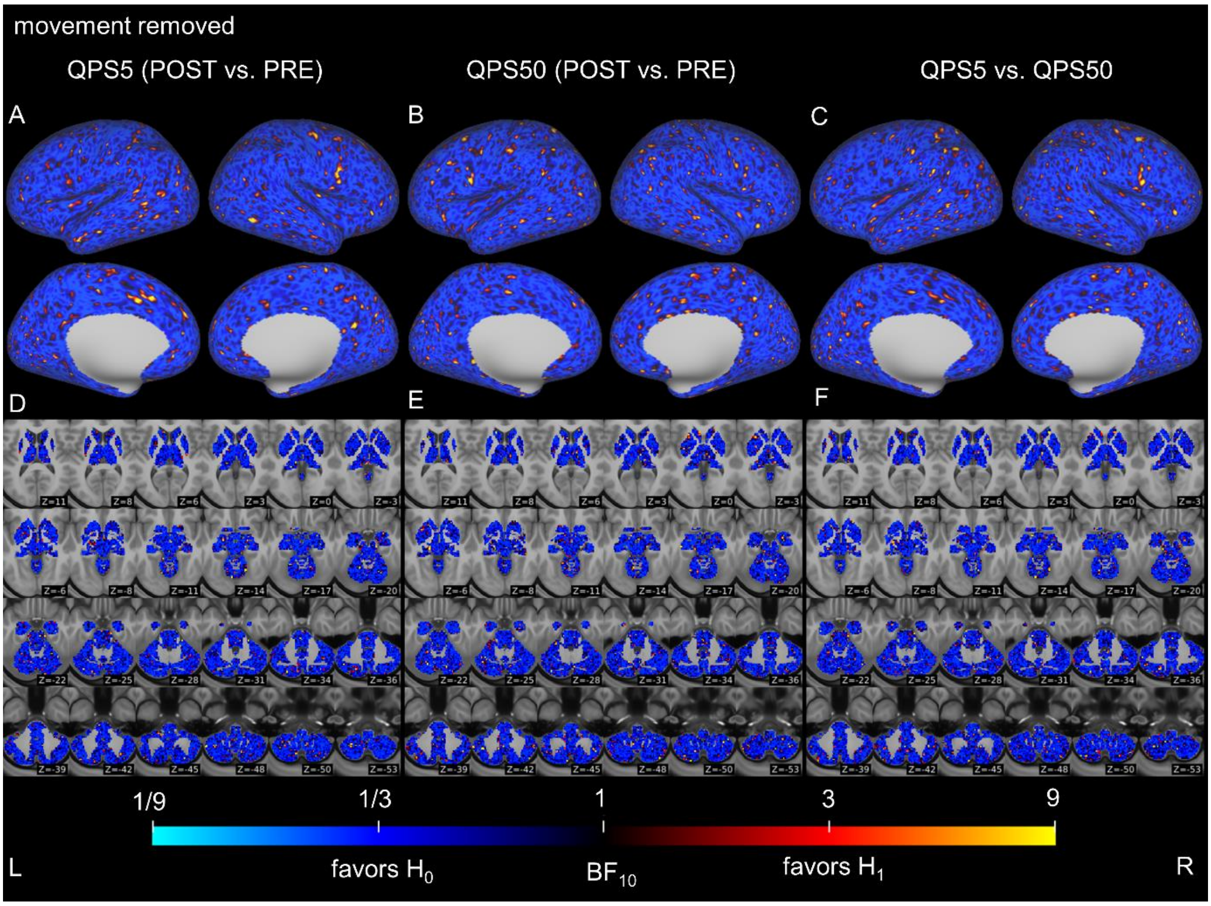

Supplementary Figure 1. Differences in fractional anisotropy (FA) values of the cerebral cortex and subcortical regions between pre- and post-QPS in the QPS5 (A and D) and QPS50 (B and E) conditions, and those in changes in FA values across conditions (C and F) after regressing out mean absolute and relative head motions. The upper panels (A–C) show the result of surface-based analysis on the cerebral cortex, whereas the lower panels (D–F) show the result of voxel-based analysis in the subcortical regions. Areas in blue indicate that the Bayes factor is less than 1 (relatively supporting the hypothesis that the effect size was zero [H_0]) when comparing FA values before and after QPS or changes in the FA values between conditions. In contrast, areas in yellow show higher than 1 (relatively supporting the hypothesis that the effect size was not zero [H_1]). Axial slices in (D–F) are shown in accordance with neurological conventions (the left side of the image represents the left side of the brain) and are displayed in the MNI coordinates from z = 11 (top left) to z = -53 (bottom right).

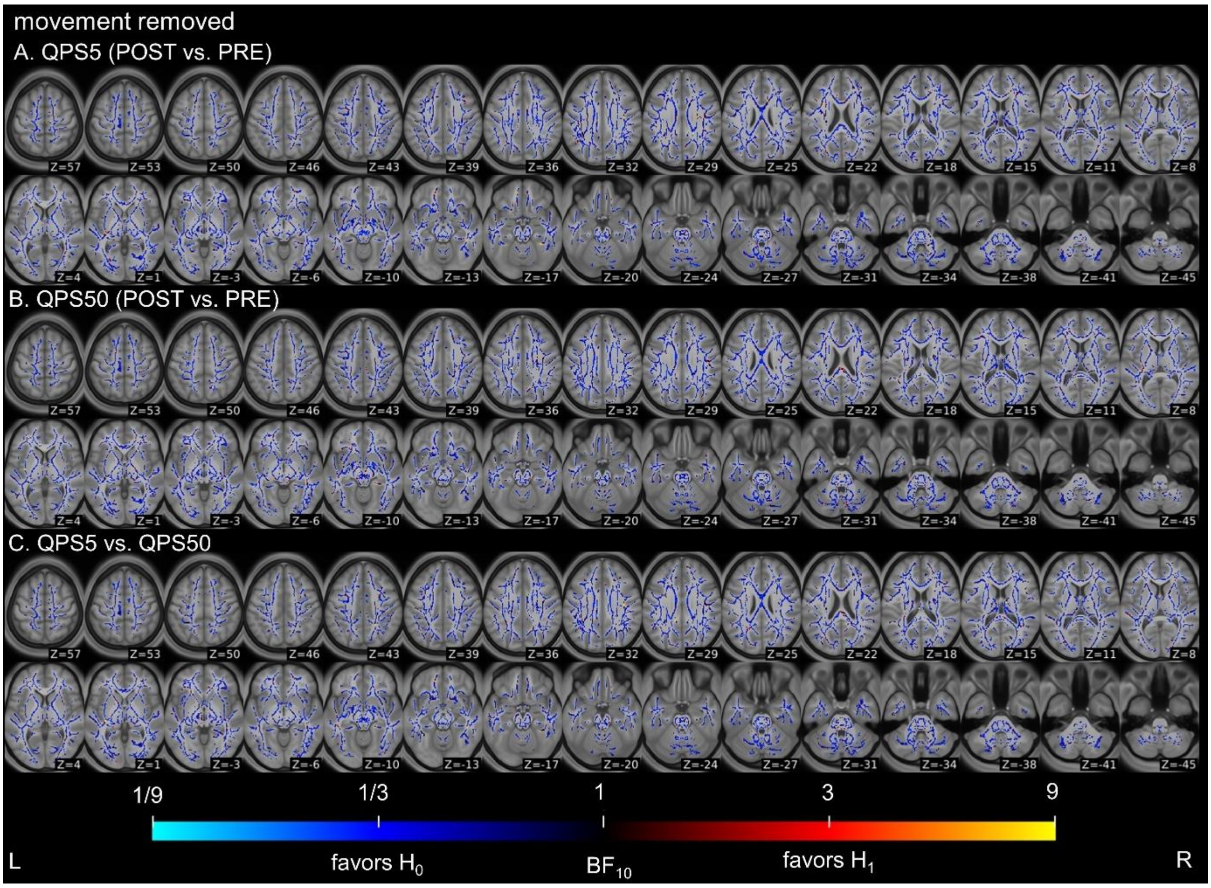

Supplementary Figure 2. Differences in fractional anisotropy (FA) values of the white matter between pre- and post-QPS in QPS5 (A) and QPS50 (B) conditions, and in the change of FA values across conditions (C), after regressing out mean absolute and relative head motions. Areas in blue indicate that the Bayes factor is less than 1 (relatively supporting the hypothesis that the effect size was zero [H_0]) when comparing FA values before and after QPS or changes in the FA values between conditions. In contrast, areas in yellow show higher than 1 (relatively supporting the hypothesis that the effect size was not zero [H_1]). Axial slices are shown in accordance with neurological conventions (the left side of the image represents the left side of the brain) and are displayed in the MNI coordinates from z = 57 (top left) to z = -45 (bottom right).

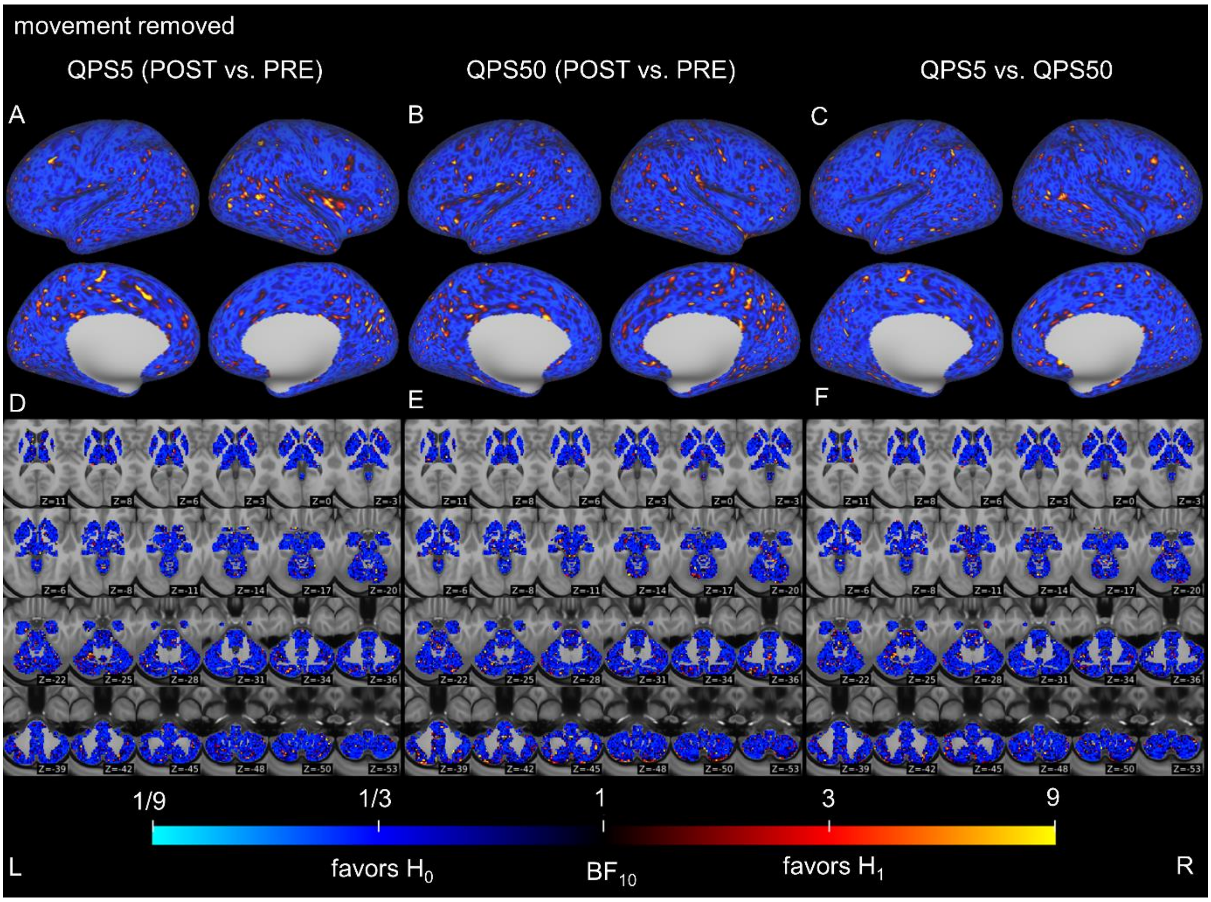

Supplementary Figure 3. Differences in mean diffusivity (MD) values of the cerebral cortex and subcortical regions between pre- and post-QPS in the QPS5 (A and D) and QPS50 (B and E) conditions, and those in changes in MD values across conditions (C and F) after regressing out mean absolute and relative head motions. The upper panels (A–C) show the result of surface-based analysis on the cerebral cortex, whereas the lower panels (D–F) show the result of voxel-based analysis in the subcortical regions. Areas in blue indicate that the Bayes factor is less than 1 (relatively supporting the hypothesis that the effect size was zero [H_0]) when comparing MD values before and after QPS or changes in the MD values between conditions. In contrast, areas in yellow show higher than 1 (relatively supporting the hypothesis that the effect size was not zero [H_1]). Axial slices in (D–F) are shown in accordance with neurological conventions (the left side of the image represents the left side of the brain) and are displayed in the MNI coordinates from z = 11 (top left) to z = -53 (bottom right).

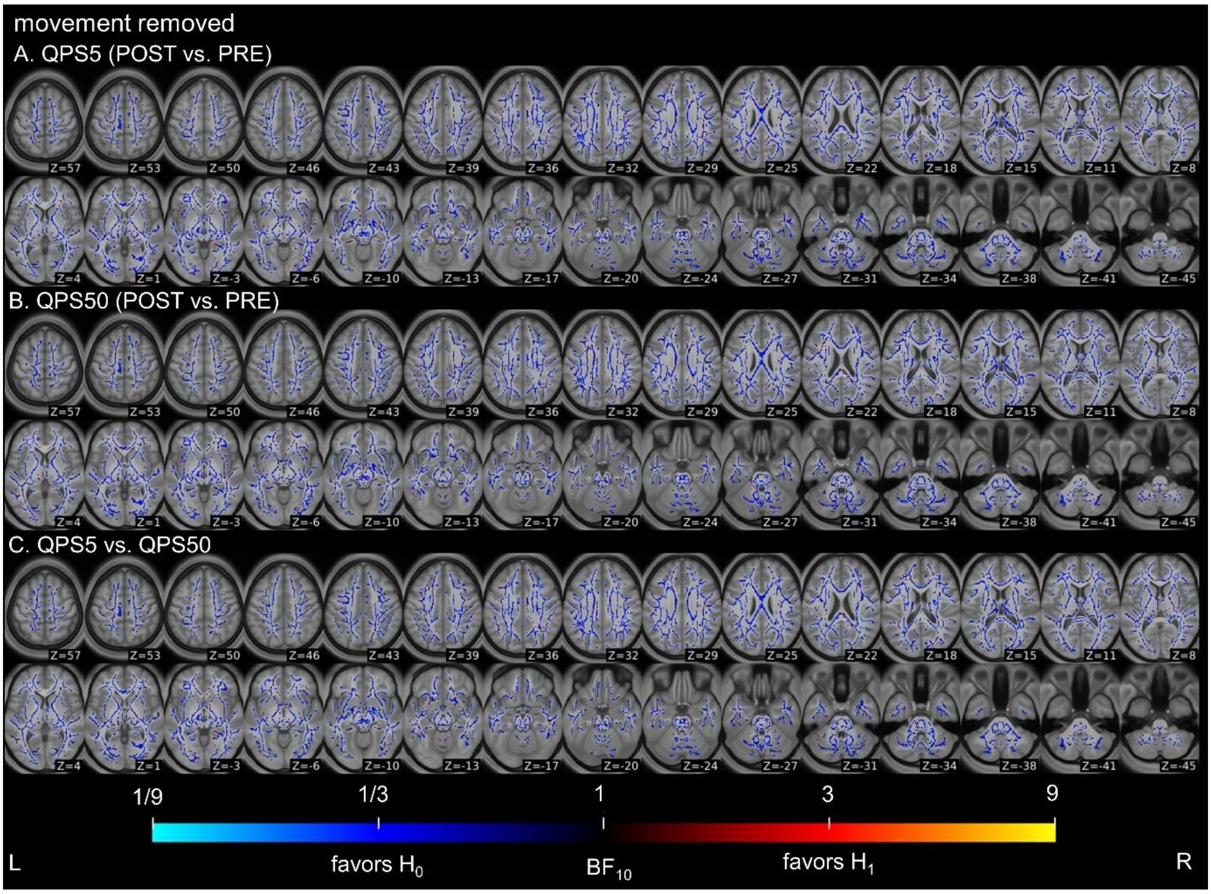

Supplementary Figure 4. Differences in mean diffusivity (MD) values of the white matter between pre- and post-QPS in QPS5 (A) and QPS50 (B) conditions, and in the change of MD values across conditions (C), after regressing out mean absolute and relative head motions. Areas in blue indicate that the Bayes factor is less than 1 (relatively supporting the hypothesis that the effect size was zero [H_0]) when comparing MD values before and after QPS or changes in the MD values between conditions. In contrast, areas in yellow show higher than 1 (relatively supporting the hypothesis that the effect size was not zero [H_1]). Axial slices are shown in accordance with neurological conventions (the left side of the image represents the left side of the brain) and are displayed in the MNI coordinates from z = 57 (top left) to z = -45 (bottom right).

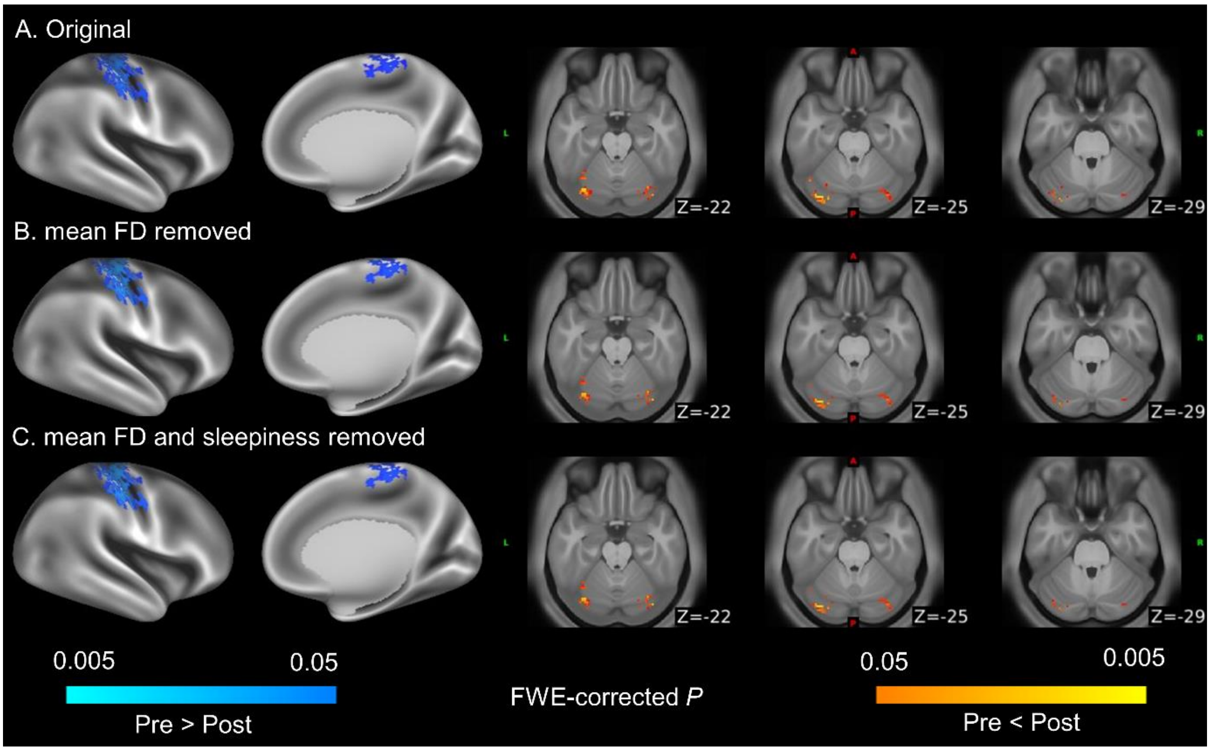

Supplementary Figure 5. Significant changes in the functional connectivity (FC) of the left M1 (i.e., the stimulated region) after QPS5 before (A) and after regressing out mean framewise displacement (FD; B) or mean FD and sleepiness (C). Areas in blue show that the FC of the left M1 was significantly decreased after QPS5, while areas in yellow indicate that it was significantly increased. Axial slices are shown in accordance with neurological conventions (the left side of the image is of the left of the brain) and are displayed in the MNI coordinates from z = -22 (top left) to z = -29 (bottom right).

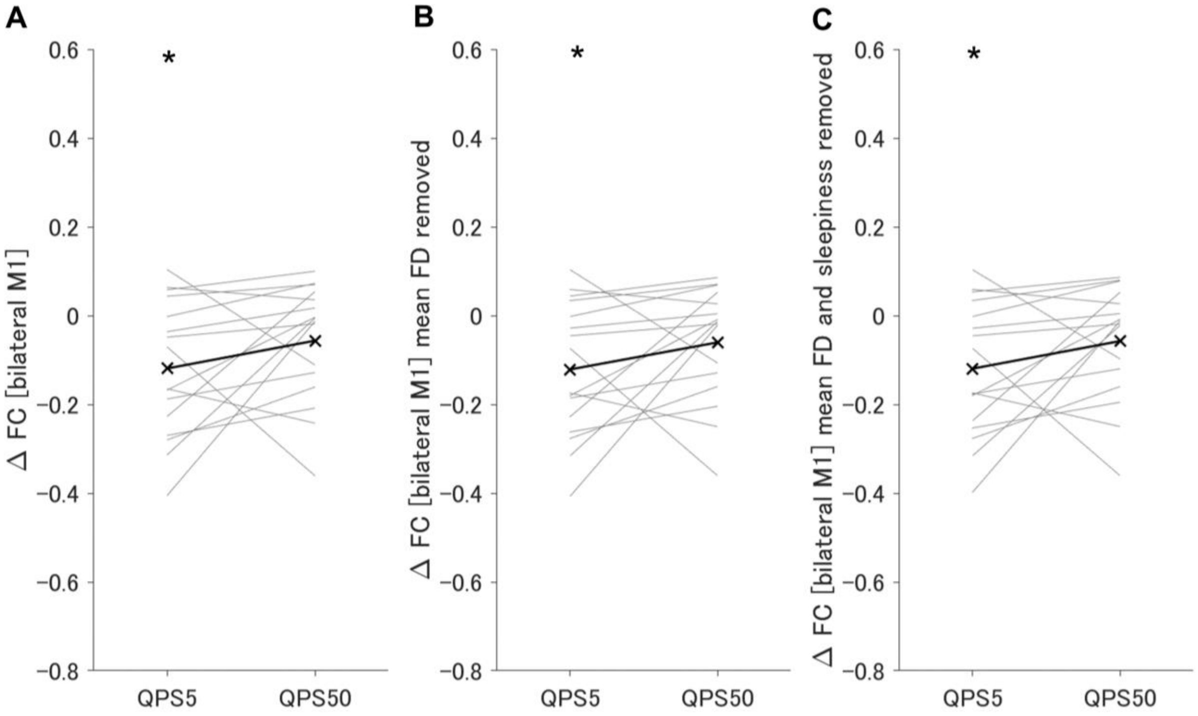

Supplementary Figure 6. Changes in the functional connectivity (FC) between the left and right M1 after QPS before (A) and after regressing out mean framewise displacement (FD; B) or mean FD and sleepiness (C). Thick black crosses indicate the mean FC change across participants. The figure on the left denotes the change after QPS5, whereas that on the right denotes the change after QPS50. The y-axis indicates the change in the FC after QPS. * shows that the change was statistically significant ($P < 0.05$).

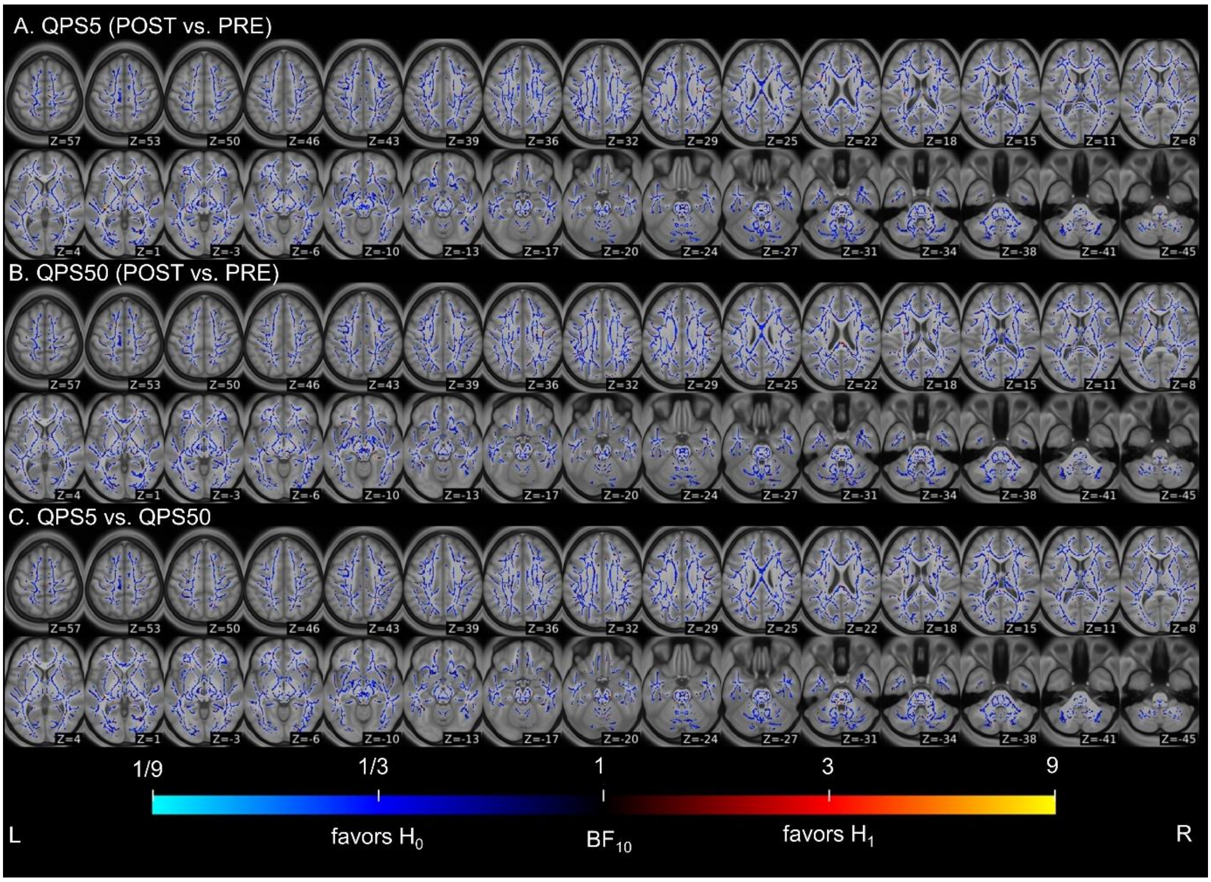

Supplementary Figure 7. Differences in fractional anisotropy (FA) values of the white matter between pre- and post-QPS in QPS5 (A) and QPS50 (B) conditions, and in the change of FA values across conditions (C). Areas in blue indicate that the Bayes factor was less than 1 (relatively supporting the hypothesis that the effect size was zero [H_0]) when comparing FA values before and after QPS or changes in the FA values between conditions. In contrast, areas in yellow show higher than 1 (relatively supporting the hypothesis that the effect size was not zero [H_1]). Axial slices are shown in accordance with neurological conventions (the left side of the image represents the left side of the brain) and are displayed in the MNI coordinates from z = 57 (top left) to z = -45 (bottom right).

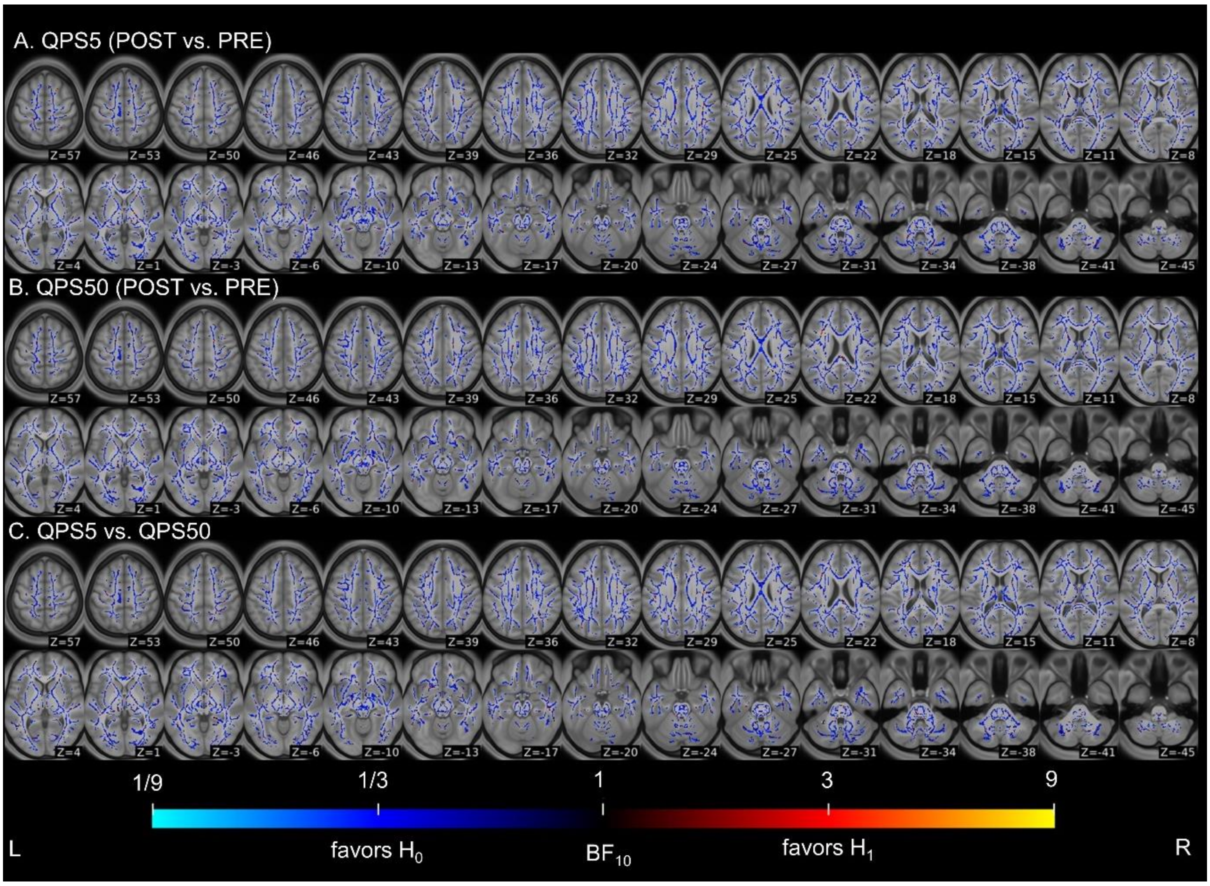

Supplementary Figure 8. Differences in mean diffusivity (MD) values of the white matter between pre- and post-QPS in QPS5 (A) and QPS50 (B) conditions, and in the change of fractional anisotropy (FA) values across conditions (C). Areas in blue indicate that the Bayes factor was less than 1 (relatively supporting the hypothesis that the effect size was zero [H_0]) when comparing FA values before and after QPS or changes in the FA values between conditions. In contrast, areas in yellow show higher than 1 (relatively supporting the hypothesis that the effect size was not zero [H_1]). Axial slices are shown in accordance with neurological conventions (the left side of the image represents the left side of the brain) and are displayed in the MNI coordinates from z = 57 (top left) to z = -45 (bottom right).

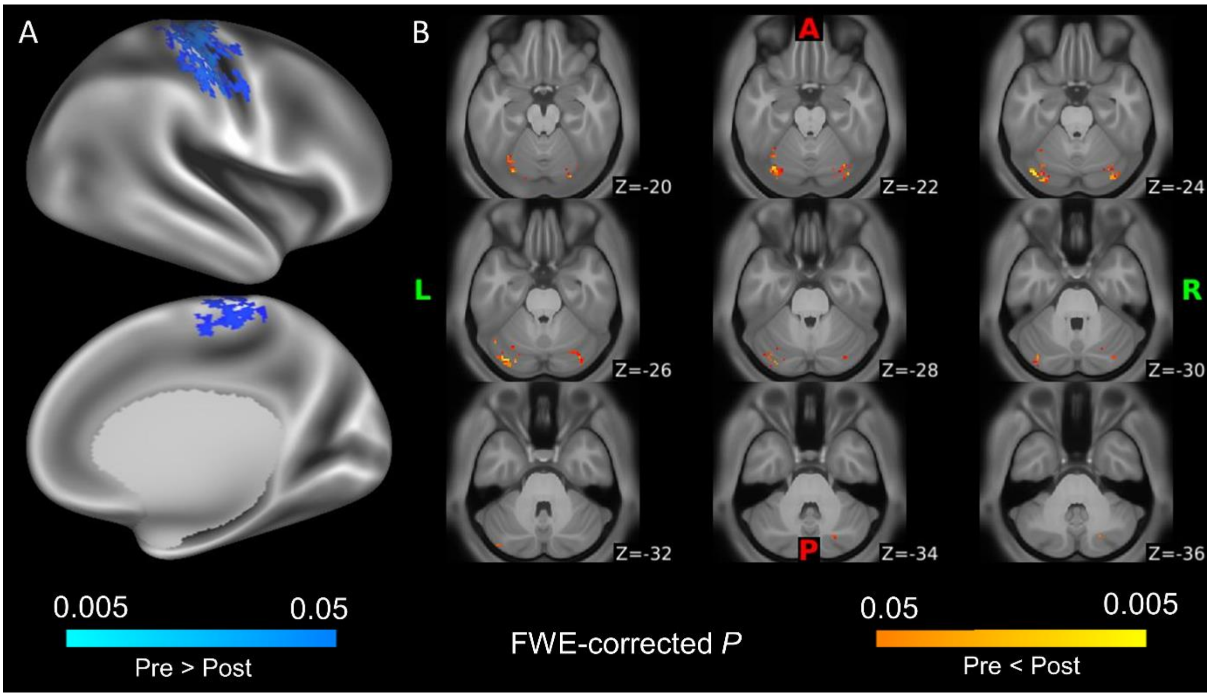

Supplementary Figure 9. Differences in the functional connectivity (FC) of the left M1 (i.e., the stimulated region) between pre- and post-QPS in QPS5 condition. (A) show the results of differences with surface-based analysis in the cerebral cortex, while (B) indicate those with voxel-based analysis in subcortical regions. Areas in blue show that the FC of the left M1 was significantly decreased after QPS5, while areas in yellow indicate that it was significantly increased. Axial slices are shown in accordance with neurological conventions (the left side of the image is of the left of the brain) and are displayed in the MNI coordinates from z = -20 (top left) to z = -36 (bottom right).

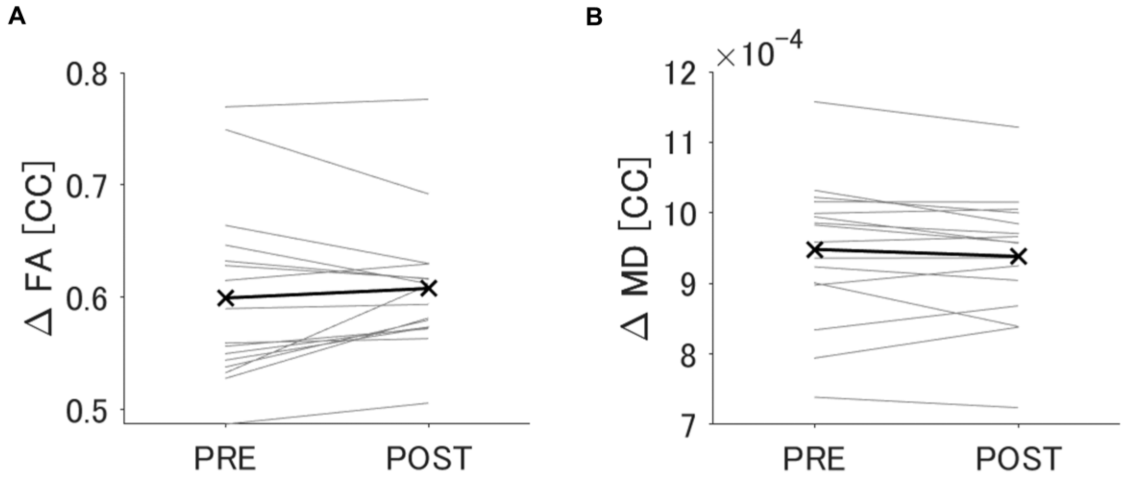

Supplementary Figure 10. Changes in the fractional anisotropy (FA; A) and mean diffusivity (MD; B) values after QPS5 in the corpus callosum (CC) connecting between the bilateral primary motor cortex. Thick black crosses indicate the mean value across participants. Each figure on the left denotes the value before QPS5, whereas that on the right denotes after QPS5.

References

Bastiani, M., Cottaar, M., Fitzgibbon, S. P., Suri, S., Alfaro-Almagro, F., Sotiropoulos, S. N., Jbabdi, S., & Andersson, J. L. R. (2019). Automated quality control for within and between studies diffusion MRI data using a non-parametric framework for movement and distortion correction. *NeuroImage*, *184*, 801–812. https://doi.org/10.1016/j.neuroimage.2018.09.073

Glasser, M. F., Coalson, T. S., Robinson, E. C., Hacker, C. D., Harwell, J., Yacoub, E., Ugurbil, K., Andersson, J., Beckmann, C. F., Jenkinson, M., Smith, S. M., & Van Essen, D. C. (2016). A multi-modal parcellation of human cerebral cortex. *Nature*, *536*(7615), 171–178. https://doi.org/10.1038/nature18933

Glasser, M. F., & Van Essen, D. C. (2011). Mapping human cortical areas in vivo based on myelin content as revealed by T1- and T2-weighted MRI. *The Journal of Neuroscience: The Official Journal of the Society for Neuroscience*, *31*(32), 11597–11616. https://doi.org/10.1523/JNEUROSCI.2180-11.2011

Griffanti, L., Douaud, G., Bijsterbosch, J., Evangelisti, S., Alfaro-Almagro, F., Glasser, M. F., Duff, E. P., Fitzgibbon, S., Westphal, R., Carone, D., Beckmann, C. F., & Smith, S. M. (2017). Hand classification of fMRI ICA noise components. *NeuroImage*, *154*, 188–205. https://doi.org/10.1016/j.neuroimage.2016.12.036

Griffanti, L., Salimi-Khorshidi, G., Beckmann, C. F., Auerbach, E. J., Douaud, G., Sexton, C. E., Zsoldos, E., Ebmeier, K. P., Filippini, N., Mackay, C. E., Moeller, S., Xu, J., Yacoub, E., Baselli, G., Ugurbil, K., Miller, K. L., & Smith, S. M. (2014). ICA-based artefact removal and accelerated fMRI acquisition for improved resting state network imaging. *NeuroImage*, *95*, 232–247. https://doi.org/10.1016/j.neuroimage.2014.03.034

Kimura, I., Ugawa, Y., Hayashi, M. J., & Amano, K. (2022). Quadripulse stimulation: A replication study with a newly developed stimulator. *Brain Stimulation*, *15*(3), 579–581. https://doi.org/10.1016/j.brs.2022.03.006

Oldham, S., Arnatkevic Iūtė, A., Smith, R. E., Tiego, J., Bellgrove, M. A., & Fornito, A. (2020). The efficacy of different preprocessing steps in reducing motion-related confounds in diffusion MRI connectomics. *NeuroImage*, *222*, 117252. https://doi.org/10.1016/j.neuroimage.2020.117252

Power, J. D., Mitra, A., Laumann, T. O., Snyder, A. Z., Schlaggar, B. L., & Petersen, S. E. (2014). Methods to detect, characterize, and remove motion artifact in resting state fMRI. *NeuroImage*, *84*, 320–341. https://doi.org/10.1016/j.neuroimage.2013.08.048

Robinson, E. C., Garcia, K., Glasser, M. F., Chen, Z., Coalson, T. S., Makropoulos, A., Bozek, J., Wright, R., Schuh, A., Webster, M., Hutter, J., Price, A., Cordero Grande, L., Hughes, E., Tusor, N., Bayly, P. V., Van Essen, D. C., Smith, S. M., Edwards, A. D., … Rueckert, D. (2018). Multimodal surface matching with higher-order smoothness constraints. *NeuroImage*, *167*, 453–465. https://doi.org/10.1016/j.neuroimage.2017.10.037

Robinson, E. C., Jbabdi, S., Glasser, M. F., Andersson, J., Burgess, G. C., Harms, M. P., Smith, S. M., Van Essen, D. C., & Jenkinson, M. (2014). MSM: a new flexible framework for Multimodal Surface Matching. *NeuroImage*, *100*, 414–426. https://doi.org/10.1016/j.neuroimage.2014.05.069

Tournier, J.-D., Smith, R., Raffelt, D., Tabbara, R., Dhollander, T., Pietsch, M., Christiaens, D., Jeurissen, B., Yeh, C.-H., & Connelly, A. (2019). MRtrix3: A fast, flexible and open software framework for medical image processing and visualisation. *NeuroImage*, *202*, 116137. https://doi.org/10.1016/j.neuroimage.2019.116137
